# Successful use of single-port laparoscopic surgery for ovarian cyst removal during pregnancy: a case series of three cases

**DOI:** 10.1093/jscr/rjad345

**Published:** 2023-06-20

**Authors:** Akihiko Misawa, Miki Muto Okubo, Seika Nagae, Ryo Yokomizo, Hiroaki Aoki, Hiroko Takanashi

**Affiliations:** Department of Obstetrics and Gynecology, Chigasaki Municipal Hospital, Chigasaki-City, Kanagawa, Japan; Department of Obstetrics and Gynecology, Chigasaki Municipal Hospital, Chigasaki-City, Kanagawa, Japan; Department of Obstetrics and Gynecology, Chigasaki Municipal Hospital, Chigasaki-City, Kanagawa, Japan; Department of Obstetrics and Gynecology, Chigasaki Municipal Hospital, Chigasaki-City, Kanagawa, Japan; Department of Obstetrics and Gynecology, Chigasaki Municipal Hospital, Chigasaki-City, Kanagawa, Japan; Department of Obstetrics and Gynecology, Chigasaki Municipal Hospital, Chigasaki-City, Kanagawa, Japan

**Keywords:** single-port laparoscopic surgery, ovarian cyst, pregnancy, laparoscopic surgery

## Abstract

Pregnant patients have an increased risk of torsion compared to that seen in nonpregnant patients, and those with larger cysts undergo torsion more frequently, which can cause obstructions during labor. The risks associated with emergent surgery are higher than those with elective surgery. Laparoscopic surgery can be safely performed during pregnancy. Single-port laparoscopic surgery is reported to be a minimally invasive laparoscopic technique. We report three cases of ovarian dermoid cysts, which were successfully removed during pregnancy through elective single-port laparoscopic surgery. In all cases, imaging showed a dermoid cyst and the cyst size was greater than 6 cm. All patients requested the surgery. The ovarian cysts were successfully removed by single-port laparoscopy without additional ports and without intra- or postoperative complications. All pregnancies progressed well and delivered vaginally at full term. The single-port laparoscopic approach is useful for removing ovarian cysts during pregnancy.

## INTRODUCTION

Approximately, 0.3–5.4% of ovarian cysts are detected during pregnancy [1], about half of which are functional or lutein cysts. While most (65.4%) of these ovarian tumors are asymptomatic, torsion occurs in approximately 8% of cases [2]. In general, symptomatic ovarian cysts and those larger than 6 cm, which are prone to torsion, are good indications for surgery. Mature cystic teratomas are the most common type of ovarian tumors requiring surgery during pregnancy, followed by serous cystadenomas, endometrioid cysts and malignant tumors [1]. Laparoscopic surgery can be safely performed during any trimester of pregnancy. Single-port laparoscopic surgery reduces postoperative pain and the need for analgesics, and it is thought to be advantageous during pregnancy. We report three cases of successful removal of ovarian cysts during pregnancy by single-port laparoscopic surgery.

## CASE SERIES

### Case 1

**Figure 1:**
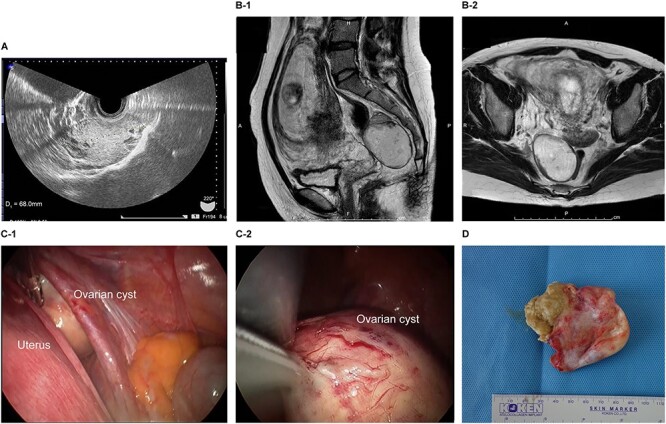
Imaging studies and laparoscopic views and specimen photograph of case 1. (**A**) Transvaginal ultrasound image showing an ovarian cyst. (**B**-1) Sagittal T2-weighted magnetic resonance image (MRI) showing an ovarian cyst. (**B**-2) Axial T2-weighted MRI showing an ovarian cyst. (**C-**1) Gestational uterus: ovarian cyst was moved from the pouch of Douglas. (**C-**2) Ovarian cyst was moved and located on the gestational uterus. (**D**) Ovarian Cyst.

The patient was a 38-year-old, gravida 3, para 1 woman with no familial or medical history. She conceived naturally and was diagnosed at 7 weeks of gestation in another medical facility. She was subsequently referred to our hospital for further examination and operation. Transvaginal ultrasonography (TVUS) revealed a fetus in the uterus and a mass 7 cm in diameter on the right ovary. Magnetic resonance imaging (MRI) revealed right ovarian cystic tumors with fat tissue. The patient’s laboratory values, including levels of tumor markers (e.g. carcinoembryonic antigen, CEA; cancer antigen 125, CA 125; cancer antigen19–9, CA 19–9 and squamous cell carcinoma antigen, SCC), were within the normal limits. The tumor was diagnosed pre-operatively as a dermoid cyst. We performed single-port laparoscopic surgery at 16 weeks and 2 days gestation to remove the right ovarian cysts. The total procedure was performed within 55 minutes, and there were no complications (Fig. 1).

### Case 2

**Figure 2:**
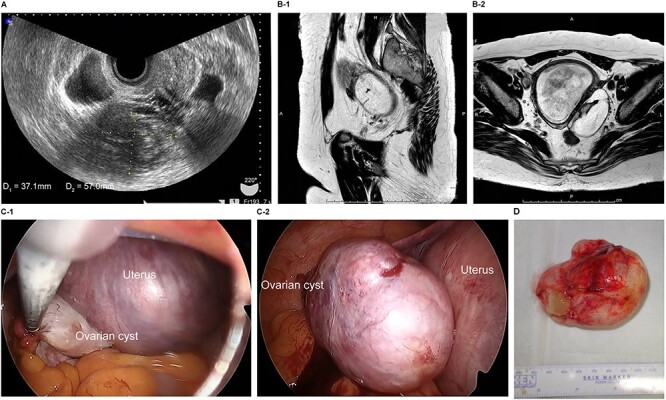
Imaging studies and laparoscopic views and specimen photograph of case 2. (**A**) Transvaginal ultrasound image showing an ovarian cyst. (**B**-1) Sagittal T2-weighted magnetic resonance image (MRI) showing an ovarian cyst. (**B**-2) Axial T2-weighted MRI showing an ovarian cyst. (**C**-1) Gestational uterus: ovarian cyst was moved from the pouch of Douglas. (**C**-2) Ovarian cyst was moved and located on the gestational uterus. (**D**) Ovarian Cyst.

The patient was a 40-year-old, gravida 1, para 0 woman with no familial or medical history. She conceived naturally and was diagnosed with a left ovarian cyst at 5 weeks gestation by another doctor. She was referred to our hospital for further examination and operation. TVUS revealed a fetus in the uterus and a mass 6 cm in diameter on the left ovary, which appeared to be a dermoid cyst. MRI revealed left ovarian cystic tumors with fat tissue. The laboratory values, including the levels of tumor markers (e.g. CEA, CA-125, CA 19–9 and SCC) were within the normal limits. We performed single-port laparoscopic surgery at 17 weeks and 0 days gestation to remove the left ovarian cysts. The procedure was performed within 59 minutes, and there were no complications (Fig. 2).

### Case 3

**Figure 3:**
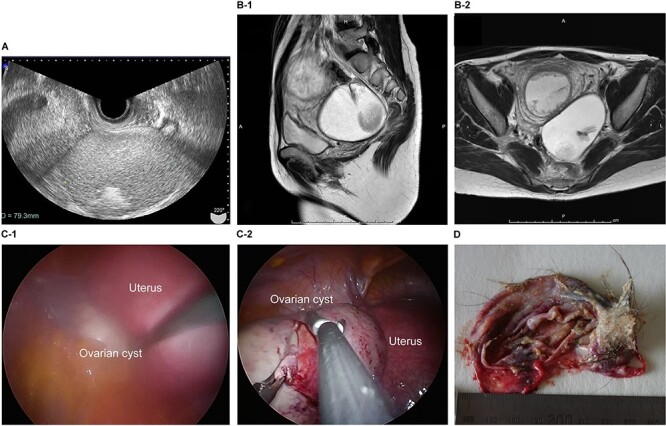
Imaging studies and laparoscopic views and specimen photograph of case 3. (**A**) Transvaginal ultrasound image showing an ovarian cyst. (**B**-1) Sagittal T2-weighted MRI showing an ovarian cyst. (**B**-2) Axial T2-weighted MRI showing an ovarian cyst. (**C**-1) Gestational uterus: ovarian cyst was moved from the pouch of Douglas. (**C**-2) Ovarian cyst was moved and located on the gestational uterus. (**D**) Ovarian Cyst.

The patient was a 29-year-old, gravida 1, para 0 woman with no familial or medical history. She conceived naturally and was diagnosed a left ovarian cyst at 7 weeks of gestation by another doctor. She was referred to our hospital for further examination and operation. TVUS revealed a fetus in the uterus and a mass 7 cm in diameter on the left ovary, which appeared to be a dermoid cyst. MRI revealed left ovarian cystic tumors with fat tissue. The laboratory values, including levels of tumor markers (e.g. CEA, CA-125, CA 19-9 and SCC) were within the normal limits. We performed single-port laparoscopic surgery at 15 weeks and 3 days gestation to remove the left ovarian cysts. The procedure was performed within 99 minutes, and there were no complications (Fig. 3).

**Figure 4:**
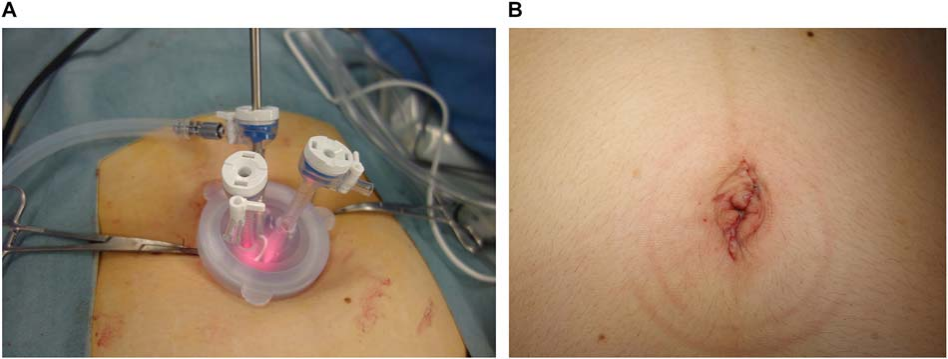
Single-port system and umbilicus wound. (**A**) Single-port system for laparoscopic viewing: Lap Disc Mini (Hakko Corporation, Osaka, Japan) and EZ Access (Hakko Corporation). (**B**) Postoperative umbilicus wound.

All patients requested elective surgery to avoid emergency surgery. For the procedure, all three patients were placed in a steep Trendelenburg position after general anesthesia was administered. Uterine manipulators were not inserted. An approximately 3-cm abdominal incision was made in the umbilicus, and the skin was retracted using a Lap Disc Mini (Hakko Corporation, Osaka, Japan) and EZ Access (Hakko Corporation). A 5-mm flexible laparoscope was used to maintain pelvic and abdominal visualization. To use conventional laparoscopic atraumatic grasping forceps, two 5-mm trocars were inserted through the Lap Disc Mini (Fig. 4). No major intra- or postoperative complications were encountered, and there was no need for conversion to multiport laparoscopic surgery. All patients were discharged from the hospital on the fourth postoperative day after an uneventful postoperative course, and the rest of the pregnancy progressed uneventfully. Each of the patients had a spontaneous vaginal delivery at term.

In all three cases, the ovarian cyst was suspected to be a dermoid cyst prior to the operation, which was confirmed by a postoperative histopathological diagnosis. The patients were cosmetically satisfied with the single scar on the umbilicus, which was difficult to recognize (Fig. 4).

## DISCUSSION

Ovarian cysts, especially during pregnancy, are being increasingly detected by ultrasound (US). Approximately, 50–70% of these cysts resolve spontaneously [3]. Asymptomatic cysts that are <6 cm in diameter are generally benign and may be managed conservatively with close US follow-up [4]. However, reported rates of torsion range from 3 to 12% in cysts with a mean size of 10 cm; furthermore, obstruction of labor has been reported in 3% of cases with cysts >3 cm, and 2–10% of patients required hospitalization for pain [5]. Pregnant patients have a 1% increased risk of torsion compared to nonpregnant patients [4]. As the rates of fetal loss and preterm birth are higher with emergent surgery than that with elective surgery (5 vs. 1% and 12 vs. 4%, respectively) [5], elective treatment is preferred in patients at risk for rupture, torsion, acute abdomen and labor obstruction [4]. According to the Society of American Gastrointestinal and Endoscopic Surgeons, laparoscopy can be safely performed during any trimester of pregnancy [6]. However, patients must be diagnosed with benign or malignant tumors by US, MRI and tumor markers, as in our cases.

Laparoscopic surgery during pregnancy for ovarian cysts may reduce the length of hospital stay without increasing the risk of miscarriage or premature delivery [5, 7]. Moreover, single-port laparoscopic surgery can be safely performed without causing additional perioperative danger, economic burden or adverse maternal and neonatal outcomes, while possibly providing increased cosmetic satisfaction [8, 9]. Our institution routinely performs single-port laparoscopic surgery for benign ovarian tumors, and the patients in our case studies did not require additional ports, were cosmetically satisfied, and experienced no complications. However, uterine operations are difficult to perform during pregnancy, even with the conventional 3- or 4-port technique. As the umbilical single-port approach allows for laparoscopic-assisted ovarian cystectomy using the umbilical wound, it may be effective for ovarian cystectomy during pregnancy, especially for patients with larger cysts or who are undergoing the operation later in pregnancy. The use of single-port laparoscopic surgery avoid emergency operation, and each of the patients had a spontaneous vaginal delivery at term; patient satisfaction was improved.

We plan to perform more single-port laparoscopic surgeries during pregnancy and will investigate the safety and surgical limitations of this method.

## CONCLUSION

We performed single-port laparoscopic surgery to remove ovarian cysts during pregnancy. All three cases were successfully completed without intra- or postoperative complications. Single-port laparoscopic surgery via the umbilical approach may be a viable treatment option for benign ovarian tumors diagnosed during pregnancy.

## Data Availability

All data generated or analyzed during this study were included in this published article.
